# Dose-Dependent Increase in Unconjugated Cinnamic Acid Concentration in Plasma Following Acute Consumption of Polyphenol Rich Curry in the Polyspice Study

**DOI:** 10.3390/nu10070934

**Published:** 2018-07-20

**Authors:** Sumanto Haldar, Sze Han Lee, Jun Jie Tan, Siok Ching Chia, Christiani Jeyakumar Henry, Eric Chun Yong Chan

**Affiliations:** 1 Clinical Nutrition Research Centre, Agency for Science, Technology and Research, Singapore Institute for Clinical Sciences, Singapore 117599, Singapore; sumanto_haldar@sics.a-star.edu.sg (S.H.); smileychiasc@gmail.com (S.C.C.); 2 Department of Pharmacy, National University of Singapore, Singapore 117543, Singapore; lee.sze.han@u.nus.edu (S.H.L.); nicholasjunjie92@gmail.com (J.J.T.); 3 Department of Biochemistry, National University of Singapore, Singapore 117596, Singapore

**Keywords:** spices, polyphenols, phenolic/aromatic acids, metabolites, nutrikinetics

## Abstract

Spices that are rich in polyphenols are metabolized to a convergent group of phenolic/aromatic acids. We conducted a dose-exposure nutrikinetic study to investigate associations between mixed spices intake and plasma concentrations of selected, unconjugated phenolic/aromatic acids. In a randomized crossover study, 17 Chinese males consumed a curry meal containing 0 g, 6 g, and 12 g of mixed spices. Postprandial blood was drawn up to 7 h at regular intervals and plasma phenolic/aromatic acids were quantified via liquid chromatography tandem mass spectrometry (LC-MS/MS). Cinnamic acid (CNA, *p* < 0.0001) and phenylacetic acid (PAA, *p* < 0.0005) concentrations were significantly increased with mixed spices consumption, although none of the other measured phenolic/aromatic acids differ significantly between treatments. CNA displayed a high dose-exposure association (R^2^ > 0.8, *p* < 0.0001). The adjusted mean area under the plasma concentration-time curve until 7 h (AUC_0–7 h_) for CNA during the 3 increasing doses were 8.4 ± 3.4, 376.1 ± 104.7 and 875.7 ± 291.9 nM.h respectively. Plasma CNA concentration may be used as a biomarker of spice intake.

## 1. Introduction

There is an increasing interest in the health effects of spices, which are widely used throughout Asia and the world to impart flavor, color and aroma within Asian cuisine. Spices have also been utilized as therapeutic applications both within the Indian Ayurveda and in Traditional Chinese Medicines over generations. The benefits of spice consumption to human health are thought to arise due to their high polyphenol contents, including several flavonoids, lignans and phenolic acids [1,2]. In recent times, there has been overwhelming epidemiological evidence supporting the association of dietary polyphenol intake with the risk reduction of several chronic diseases, including type 2 diabetes, cardiovascular diseases, neurodegenerative diseases, and some cancers. The elucidation of specific bioactive phytochemicals responsible for these beneficial effects is paramount in understanding the mechanisms for their protective effects. While parent polyphenols often display desirable effects in vitro, their relevance is somewhat limited in vivo, given their low oral bioavailability and low systemic concentrations [3]. On the other hand, bioactive metabolites derived from the extensive in vivo biotransformation of dietary polyphenols may exhibit higher systemic exposure, but often display large interindividual differences [4]. It is therefore pertinent to conduct bioavailability studies [5] in a real life dietary context, to characterize the systemic exposure of polyphenolic metabolites so as to elucidate the biological effects of the parent polyphenols [1].

Several authors have provided extensive overviews on the metabolic fate of plant polyphenols upon consumption in human diet [6,7,8]. In summary, some dietary polyphenols which are consumed as glycosides are hydrolyzed to their aglycone metabolites in the small intestine prior to absorption. They may eventually be conjugated to form methyl/sulfate/glucuronide metabolites in the liver prior to their systemic absorption and disposition. However, glycosides that are incompletely absorbed in the small intestine can transit to the large intestine, where they are metabolized by the microflora in the colon. A diverse group of dietary polyphenols and their metabolites are catabolized into a relatively small number of terminal metabolites, principally phenolic/aromatic acids. Therefore, the systemic exposures of dietary parent polyphenols are often limited [9,10]. Consequently, the quantity and identities of downstream metabolites of dietary polyphenols are relatively similar, irrespective of the dietary source of polyphenols [11]. Therefore, studies exploring bioefficacy of polyphenols need to take this fact into account [12].

Phenolic acids are one of the major byproducts of the metabolism of several polyphenols. While foods such as fruits, vegetables, tea and coffee are important sources of dietary phenolic/aromatic acids in the Western diet [13], several spices and base vegetables such as ginger, onions etc. used in curries are also rich sources of phenolic/aromatic acids [2] within an Asian dietary context. Given that phenolic/aromatic acids per se are highly bioavailable [14], these compounds may in fact be largely responsible the beneficial effects seen with consumption of spices [15,16]. However, to our best knowledge, there have been no randomized controlled dose-response trial that has specifically investigated the bioavailability of phenolic/aromatic acids from dietary doses of mixed spices, as normally eaten in curries.

We have recently undertaken the Polyspice Study, which found significant improvements in glucose homeostasis in response to increasing intake of polyphenol rich curry made with 7 mixed spices and 4 base vegetables [17], as well as observing dose-dependent increases in postprandial plasma glucagon-like peptide 1 (GLP-1) concentration [18]. Given that phenolic/aromatic acids and/or their derivatives in systemic circulation have been shown to modulate glucose homeostasis [19] as well as increase postprandial GLP-1 response [20], the primary aim of this present study was to further investigate whether the dose-dependent increases in the consumption of polyphenol rich mixed spices and base vegetables can lead to increases in plasma concentrations of a panel of phenolic/aromatic acids. The panel of phenolic/aromatic acids chosen for analyses were based on the native phenolic/aromatic acids present in the test meal ingredients [2], as well as by predicting the metabolic fate of the parent polyphenols (e.g., various flavonoids, phenolic/aromatic acids, lignans, and curcuminoids) present in these ingredients. The potential pathways for metabolism of parent polyphenols are outlined into a metabolic map (Figure S1) [8,21]. Given their diversity, not all polyphenols would be metabolized via the outlined metabolic pathways, although we hypothesized that a substantial proportion of parent polyphenols present in the fed meal would be metabolized into a small panel of terminal phenolic/aromatic acids metabolites, plasma concentrations of which would vary according to the amounts of polyphenol consumed. Furthermore, the secondary aim of this study was to establish the plasma nutrikinetic profiles of the measured phenolic/aromatic acids in response to spice consumption at different doses. The findings of this study should therefore inform future dose-exposure-effect studies with spices and/or other polyphenol rich foods.

## 2. Materials and Methods

### 2.1. Study Design

The detailed methods for this Polyspice study have been described elsewhere [17]. In brief, this was a three-way randomized, controlled, crossover, acute feeding trial in healthy Chinese males (age 23.7 ± 2.30 y, BMI 23.0 ± 2.31 kg/m^2^). Three days prior to the main test day, each volunteer avoided vigorous physical activity and adopted a low polyphenol diet. To help adhere to a ‘low polyphenol diet’ in this run-in period, volunteers were provided with a list of polyphenol rich foods to avoid, which consisted of some fruits and fruit juices, spices and condiments, olive oil, certain vegetables, whole grain cereals, cocoa products, tea (including Green tea), coffee, red wine etc. The volunteers were also provided with suggested foods they could have, which included meat and meat products, dairy and dairy products, refined cereals, potatoes etc. On the main test day, the volunteers arrived at the study center in the morning after an overnight fasting period of a minimum of 10 h. On each of 3 test sessions, the volunteers consumed in random order 1 of 3 test meals: Dose 0 Control (D0C), Dose 1 Curry (D1C) and Dose 2 Curry (D2C). D0C, D1C, and D2C contained 0 g, 6 g, and 12 g mixed spices respectively. The mixed spices were prepared by thoroughly mixing dried powders of 7 different spices consisting of turmeric (Everest Spices, Mumbai, India), coriander seeds (Everest Spices, Mumbai,, India), cumin seeds (Everest Spices, Mumbai, India), dried Indian gooseberry (‘*amla*’, *Emblica officinalis*, Ramdev Spices, Ahmedabad, India), cayenne pepper (Robertson’s, Durban, South Africa), cinnamon (McCormick’s, Baltimore, MD, USA), and clove (Robertson’s, South Africa) in the ratio of 8:4:4:4:2:1:1 respectively. The ‘base vegetables’ used in the curry recipes consisted of tomatoes, onions, ginger, and garlic in the ratio of 5:2:1:1, with D0C, D1C and D2C containing 0 g, 90 g, and 180 g base vegetables respectively. The composition of the major polyphenols as well as their concentrations found in the spices and based vegetables used in our test meals are listed in Table S1. The 3 test meals were matched for total vegetable content by including 130 g peeled eggplant (*Solanum melongena*) and 50 g tomatoes to D0C and 90 g peeled eggplant to D1C. Plain white rice was consumed along with each test meal and the composition of all 3 test meals were isocaloric and macronutrients matched. A standardized lunch was served after the 3 h postprandial blood draw time point, consisting of a made-to-order chicken lasagna (TopChoice Food Industries (S) Pte Ltd., Singapore), which specifically excluded polyphenol-rich ingredients such as black pepper, herbs, spices, onion, garlic or ginger from the product’s original recipe. There was a minimum of 1-week washout period between each session.

The study was approved by the Domain Specific Review Board in Singapore (ref: C/2015/00729), registered at ClinicalTrial.gov (ref: NCT02599272) and was conducted in accordance with the Declaration of Helsinki and the Singapore Good Clinical Practice (GCP) guidelines. All participants provided written informed consent. In total, 17 volunteers completed all three study sessions while the remaining 3 volunteers only undertook D0C and D2 sessions.

### 2.2. Total Polyphenol Content (TPC) of Test Meals

The TPC of the prepared test meals were analyzed using the Folin-Ciocalteu assay and expressed in gallic acid equivalents (GAE), as described previously [17]. The TPC (mean ± SD) per portion (without rice) were 130 ± 18.7 mg GAE, 556 ± 19.7 mg GAE and 1113 ± 211.6 mg GAE for D0C, D1C, and D2C respectively.

### 2.3. Blood Sample Collection

Venous blood was collected into 6 mL BD Vacutainer^®^ Plus Plastic K_2_ EDTA (BD, Franklin Lakes, NJ, USA) tubes at baseline prior to test meal consumption (0 h) followed by 12 postprandial time points at regular intervals: 0.5 h, 1.0 h, 1.5 h, 2.0 h, 2.5 h, 3.0 h, 3.5 h, 4.0 h, 4.5 h, 5 h, 6 h, and 7 h. The plasma sample was centrifuged at 1500 *g* for 10 min at 4 °C within 45 min of collection. Aliquots were stored at −80 °C until analysis.

### 2.4. Plasma Phenolic/Aromatic Acids Analyses

The panel of phenolic/aromatic acids chosen for analyses were based on the predicted metabolic fate of polyphenols contained within the ingredients used in our test meals, as described above. The selected phenolic/aromatic acids and their sources were as follows: 3-(3-hydroxyphenyl)-propanoic acid (3OH-PPA), 3-hydroxyhippuric acid (3OH-HA) and 4-hydroxyhippuric acid (4OH-HA) were purchased from Carbosynth (Berkshire, UK); benzoate-d5 (BA-d5) from Cambridge Isotope Laboratories (Andover, MA); 3-(4-hydroxyphenyl)-propanoic acid (4OH-PPA), 3-phenylpropanoic acid (PPA), cinnamic acid (CNA), 3-hydroxybenzoic acid (3OH-BA), 4-hydroxybenzoic acid (4OH-BA), benzoic acid (BA), hippuric acid (HA), phenylacetic acid (PAA), 3-hydroxyphenylacetic acid (3OH-PAA), 4-hydroxyphenylacetic acid (4OH-PAA), gallic acid, and Folin-Ciocalteu reagent from Sigma-Aldrich (St. Louis, MO, USA); acetic acid from Merck (Darmstadt, FRG). Ultra-pure water from Adrona (Rīga, Latvija) was used. Unless otherwise stated, all chemicals and reagents were obtained from Sigma-Aldrich (St. Louis, MO, USA).

Stock solutions of 10 mM of each analyte were prepared in acetonitrile. Baseline (0 h) plasma was pooled from all participants and used as a matrix in equal volume for each calibration standard. BA, 3OH-BA, 4OH-BA, 3OH-PPA, 4OH-PPA, 3OH-HA, 4OH-HA, CNA were diluted to calibration concentrations of 5–1000 nM, while PAA, 3OH-PAA, 4OH-PAA, PPA, HA were diluted to concentrations of 25–5000 nM. All standard solutions were freshly prepared and kept in −20 °C prior to use.

### 2.5. UHPLC-MS/MS Analysis

Protein precipitation was undertaken using 400 μL acetonitrile containing 1 µM of BA-d5 internal standard was added to 100 μL of plasma, calibration, quality control or blank samples. The sample was centrifuged at 16,000 *g* for 15 min at 4 °C. 400 μL of the supernatant was recovered and evaporated under a nitrogen flow. Finally, extracts were reconstituted with 80 μL of 90% mobile phase A and 10% mobile phase B.

The detection of plasma phenolic acids was performed on an Agilent Infinity 1290 Liquid Chromatography System (Santa Clara, CA, USA) coupled to a SCIEX Triple Quad 3500 mass spectrometry (Vaughan, ON, Canada). 5 μL of reconstitute was injected in an ACQUITY UPLC C_18_ column, 2.1 × 100 mm, 1.7 μm (Milford, MA, USA) with an ACQUITY UPLC BEH C_18_ guard column, 2.1 × 5 mm, 1.7 μm (Milford, MA, USA). The mobile phase consisted of 0.01% acetic acid (solvent A) and acetonitrile with 0.01% acetic acid (solvent B). The elution profile (flow rate of 0.5 mL/min) started at 10% solvent B and was increased linearly from 10% to 75% between 0.5 to 3.5 min and to 100% at 3.5 min at which percentage of solvent B was held constant for 1 min. The gradient was reverted to 10% solvent B for 1 min for equilibration. Electrospray ionization was performed in the negative ionization mode (ESI-) with gas temperature 500 °C, curtain gas 30 psi, collision gas 7 psi, ion source gas 1 (60 psi), ion source gas 2 (60 psi) and capillary voltage −4500 V. The multiple reaction monitoring (MRM) conditions for each analyte were determined by direct infusion into the MS. MultiQuant software 3.0.2 (Vaughan, ON, Canada) was used to analyze the data. Optimized MS component-dependent parameters are summarized in Table S2. As analytes are endogenous in nature, quantification was performed via a method of background subtraction [22]. Accuracy and precision of the quantification are reported in Table S2.

### 2.6. Statistical Analyses

The maximum concentration in plasma (C_max_), time needed to reach C_max_ (T_max_), and area under the plasma concentration-time curve until 7 h (AUC_0–7 h_) were calculated using Phoenix WinNonlin 6.3 (Mountain View, CA, USA). Mean AUC_0–7 h_ and C_max_ was normalized against the basal levels of each individual to account for differences in inter-individual baseline plasma concentrations of the metabolites. The normalized data was evaluated for statistical differences using repeated measures one-way ANOVA analysis with post-hoc Bonferroni’s correction. Linear regression analysis between TPC of each meal and AUC_0–7 h_ was further performed. Dose-dependent linear correlation coefficients (R^2^) were determined using GraphPad Prism 6 (San Diego, CA, USA).

As CNA displayed a strong dose-exposure relation, a nonlinear least-squares nutrikinetic modeling was further performed on its plasma concentration-time profile from 0 to 7 h post consumption. Compartmental parameter estimates for CNA were determined after fitting of concentration data to several compartmental models on Phoenix WinNonlin (Table S3). The Gauss-Newton algorithm was applied and the final model selection was based on goodness-of-fit comparisons, examination of the residual plots, correlation of the observed and predicted values, and Akaike information criterion. 1/Y^2^ model yielded the best fit amongst the various weighting schemes used (1/Y, 1/Y^2^, and actual). The final nutrikinetic curve best fit into a one-compartment, first-order elimination, as described by:

(1) $$C\left(T\right)=\frac{DK_{a}}{V\;(K_{a}-K_{e})}\;\left[e^{-K_{e}T}-e^{-K_{a}T}\right]$$

where C is the plasma concentration, T is time, *K_a_* is the first-order absorption rate, *K_e_* is the first-order elimination rate constant, D is dose, and V is the apparent volume of distribution. Secondary parameters determined from the model included the C_max_, T_max_, AUC_0–7 h_ as well as the terminal half-life.

## 3. Results

**Figure 1:**
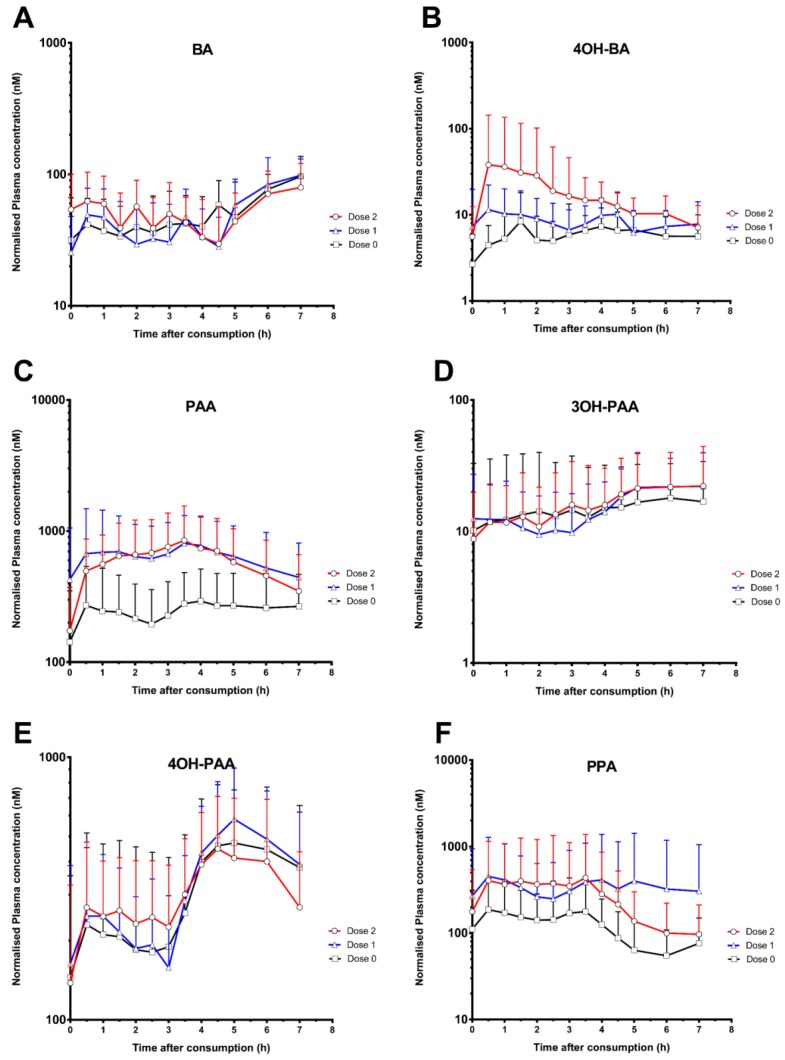

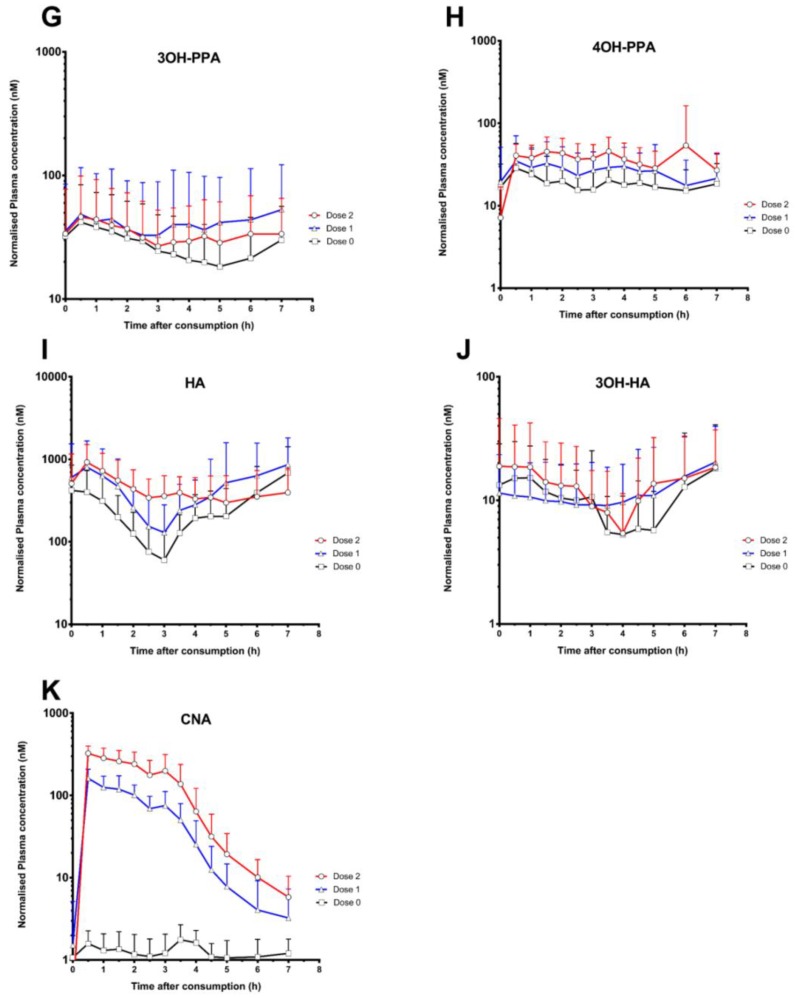
Normalized logarithmic plasma concentration-time curves of 11 phenolic/aromatic acids, specifically (**A**) benzoic acid (BA); (**B**) 4-hydroxybenzoic acid (4OH-BA); (**C**) phenylacetic acid (PAA); (**D**) 3-hydroxyphenylacetic acid (3OH-PAA); (**E**) 4-hydroxyphenylacetic acid (4OH-PAA); (**F**) 3-phenylpropanoic acid (PPA); (**G**) 3-(3-hydroxyphenyl)-propanoic acid (3OH-PPA); (**H**) 3-(4-hydroxyphenyl)-propanoic acid (4OH-PPA), (**I**) hippuric acid (HA); (**J**) 3-hydroxyhippuric acid (3OH-HA); and (**K**) cinnamic acid (CNA).

Plasma concentration-time curves of the 11 quantified metabolites across all three doses were presented in Figure 1. The C_max_, T_max_ and AUC_0–7 h_ for each metabolite are reported in Table 1. Two phenolic acids, namely 3OH-BA and 4OH-HA were found to be below detection limits. The plasma C_max_ ranged from the low nM to low µM between the metabolites, peaking at various times (T_max_).

AUC_0–7 h_ for the various phenolic/aromatic acids were calculated as a measure of the systemic exposure of the metabolites. Scatter plots between the mean normalized AUC_0–7 h_ of each phenolic/aromatic acid against the TPC of D0C, D1C and D2C meals indicated large inter-individual variations in the dose-exposure associations and also allowed visualization of outliers (Figure S2). The majority of the phenolic/aromatic acids presented a low dose-exposure correlation after linear regression analysis, apart from CNA. CNA appeared in blood plasma shortly after meal consumption and peaked approximately 1 h post consumption for D1C and D2C. However, for CNA, one particular volunteer was a significant outlier. Even though his results displayed a strong dose-exposure relationship for CNA (Figure S3), this individual had a 15-fold higher AUC_0–7 h_ than the mean CNA concentration at D2C. This individual was therefore excluded from the subsequent analysis of CNA.

A repeated measures ANOVA showed significant increases in AUC_0–7 h_ of CNA when comparing D0C to D1C to D2C (F(1.109, 16.63) = 75.94, *p* < 0.0001), with post hoc tests using Bonferroni correction revealing significant differences between D0C and D1C, between D0C and D2C, and between D1C and D2C (all *p* < 0.0001). Similarly, significant increases in AUC_0–7 h_ of PAA with the same comparisons (F(1.964, 31.42) = 9.847, *p* < 0.0005), with post hoc tests using Bonferroni correction showing significant differences between D0C and D1C (*p* < 0.001), and between D0C and D2C (*p* < 0.0054). AUC_0–7 h_ of other phenolic/aromatic acids were not statistically significantly different between various doses, even though there were some indication of increases in some phenolic/aromatic acids such as 4OH-BA during the curry sessions (D1C and D2C) as compared with the control session.

**Figure 2:**
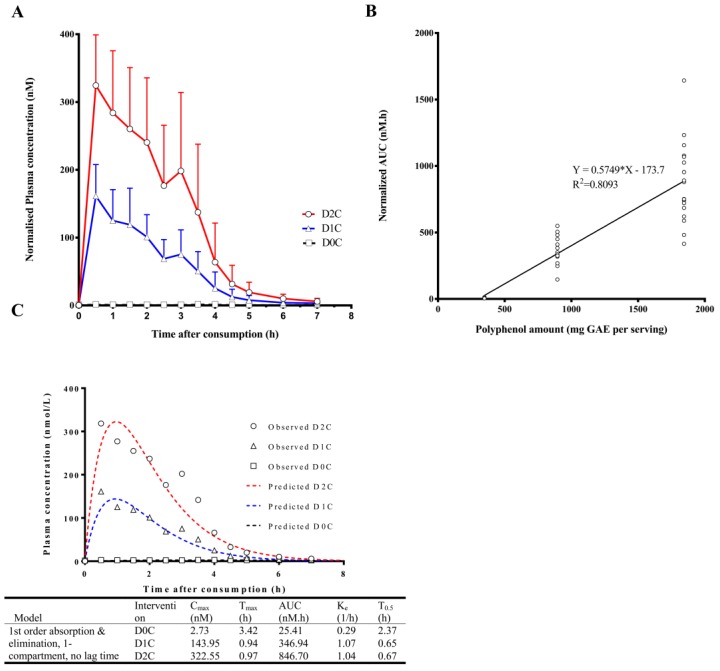
(**A**) Normalized linear plasma concentration against time plots of cinnamic acid. (**B**) Scatter plot and linear regression of normalized area under the curve (AUC) against polyphenol amount expressed in gallic acid equivalent (GAE) of cinnamic acid. (**C**) Nutrikinetic modeling of cinnamic acid, best fitted by a one-compartment model. AUC, normalized area under the curve; C_max_, normalized maximum concentration obtained in plasma (nM); K_e_, elimination rate constant; T_0.5_, half-life; T_max_, time (h) when C_max_ is obtained.

A closer examination in the nutrikinetics of CNA also revealed the presence of a secondary peak during both D1C and D2C periods at around 3 h (Figure 2A). Regression analysis of CNA AUC_0–7 h_ against the polyphenol amount expressed in GAE showed a strong linear correlation R^2^ of 0.8093 (Figure 2B). Compartmental modeling of CNA showed best fit to a no lag time, 1st order absorption, 1st order elimination, 1-compartment model, with average elimination half-life of 0.66 h and T_max_ at 0.96 h (Figure 2C). It should be noted that the calculated half-life assumes that elimination of CNA is rate-limiting. In cases where the absorption is rate-limiting (i.e., slow absorption of CNA in gastrointestinal tract into bloodstream), the observed half-life would be longer than expected (“flip-flop kinetics”). In the latter, an i.v. administration would be necessary to determine the true elimination half-life.

## 4. Discussion

While spices are known to contain high levels of polyphenols, our study is the first to show dose-dependent increases in plasma CNA concentrations in response to increasing doses of mixed spice consumption. In support of our findings, previous studies with other polyphenol rich foods such as wholegrain cereals [23], tea [9], coffee [24] and berries [10,25] have all shown increases in the plasma levels of phenolic acids upon consumption. Thus, our findings confirmed that these phenolic and aromatic acids, particularly CNA, are indeed important systemic metabolites of spices-derived dietary polyphenols.

The blood sampling regime at numerous time points and at regular intervals during a 7-h postprandial period in this study allowed for a detailed insight on the plasma nutrikinetics of the phenolic/aromatic acids in relation to the dose-dependent increases in curry intake. We restricted our analyses only to free, unconjugated phenolic/aromatic acids, without the any prior enzymatic deconjugation. This is because several uncertainties exist regarding the widespread use deconjugating enzymes (such as glucoronidases and sulfatases) in studies exploring bioavailability of polyphenols. Firstly, the deconjugating enzymes may often only partially hydrolyse the relevant phenolic/aromatic acid conjugates [26,27] which could lead to either overestimation or underestimation of phenolic/aromatic acids in vivo. Secondly, glucuronidated conjugates can spontaneously undergo deconjugation in vivo after reacting with hydroxyl or sulfhydryl groups to reform free phenolic/aromatic acids [28]. Finally, it is also known that certain polyphenolic compounds, including phenolic acids, tend to display greater bioactive potencies in their free forms than in their conjugated forms [29,30]. Individual phenolic/aromatic acids displayed a high degree of inter-individual variability in their C_max_ and systemic exposure (AUC_0–7 h_) in response across the 3 doses of curry. This was probably due to the fact that polyphenols are simultaneously metabolized both by the human (host) metabolic pathways and by the gut microflora present, which can collectively modify the rate and extent of absorption, distribution and excretion of dietary polyphenols [4].

The inter-individual variation in phenolic/aromatic acid nutrikinetics was large despite the rigorously controlled study design (Figure 1) and the careful validation of the analytical methods (Table S2). Similar extents of inter-individual variability were also observed in several other dietary intervention studies [31,32]. We postulate that the observed nutrikinetic variability might be a result of variations in host xenobiotic disposition functions including complex oral absorption and hepatic clearance mechanisms, as well as variations in gut microbial functions. Presently there are still lapses in our understanding on the roles and the relative contributions of specific bacterial species as well as differences in host single nucleotide polymorphisms (SNPs) towards this variability. Moreover, using integrated systems biology tools, our laboratory previously investigated the complex roles of gut microbiota on the systemic absorption and disposition of a xenobiotic [33]. Similar approaches can be applied in future studies to validate our postulation on the variable nutrikinetics of polyphenol metabolites.

Hippuric acid (HA) was identified as the most abundant phenolic acid in plasma with a maximum concentration reaching above 1200 nM (Table 1). We observed an initial increase in plasma HA concentration immediately after the mixed spice consumption followed by a dip below baseline followed by further increase again. While this initially appeared idiosyncratic, our results corroborated with the observations of three reports that investigated the nutrikinetics of HA [10,32,34]. We postulate the high baseline concentration of HA was associated with the consumption of non-polyphenol sources of HA prior to the study, given that the 15 h overnight fasting period during this study was shorter than the 22 h half-life of HA [35]. Moreover, the increases in HA after 5–6 h consumption indicate bacterial metabolism in the colon particularly since in vivo HA concentration has been previously shown to be associated with gut microbiome content [36]. These findings are certainly important considerations while using HA as a biomarker of total polyphenol intake.

Phenylacetic acids (PAA, 3OH-PAA, and 4OH-PAA) are often present in blood plasma after consumption of polyphenol-rich food sources [32]. While all three phenylacetic acids were detected in our study, only the systemic exposure of PAA was significantly increased after consumption of D1C and D2C meals. PAA is an endogenous product of phenylalanine metabolism circulating at low concentration levels, but is also a well-known gut microbiome metabolite derived from colonic bacterial metabolism of unabsorbed polyphenols [37]. The microbial origin of PAA is also demonstrated when it was found to be excreted in greater amount after germ-free rats were inoculated with fecal microorganisms [38]. The similar increases in plasma PAA concentrations during both D1C and D2C sessions may suggest either rate-limiting steps along the degradation of polyphenols by colonic bacteria, and/or limiting absorption processes by intestinal epithelial cells, possibly indicating saturation even at the lower mixed spice dose (D1C session).

Benzoic acids (BA, 3OH-BA, 4OH-BA) represent smaller systemic exposures relative to other aromatic acids analyzed in this study. Particularly striking was the dose-dependent increase in 4OH-BA concentration levels post-consumption (Figure 1B), which suggest the presence of the free hydroxybenzoic acid or its parent conjugate in the mixed spices consumed. A high-resolution analysis of various herbs and spices [39] also revealed a high amount of 4OH-BA in cinnamon (1.19 ± 0.04 μg/g) and cumin (1.61 ± 0.06 μg/g). Additionally, more free acids may be released from their conjugates (e.g., as glucosides) during food processing [40], or during metabolism by the gut microbiome and the host. The multiple sources of 4OH-BA may explain both its dose-dependency and the high variability observed in the participants of the study.

Cinnamaldehyde, the precursor of CNA, is a major constituent in Ceylon cinnamon and other species under the genus *Cinnamomum* [41]. Cinnamaldehyde and its derivatives have gained much interest in recent years for its ability to alleviate diabetic complications in vivo [19], and its pharmacology and pharmacokinetics have been extensively reviewed by Zhu et al. [42]. Cinnamaldehyde is not stable in the body. It was reported to be partly oxidized to CNA in the stomach and intestine [43], and extensively oxidized to CNA after absorption. Given the strong linear correlation between the normalized AUC_0–7 h_ of CNA and the oral doses of mixed spices in our study, CNA represents a robust biomarker of dietary spice consumption. The pharmacokinetic investigation of CNA in humans is currently limited and to our knowledge, there is only one previous report on the nutrikinetic profile of CNA in humans [44], which utilized a two-compartment, first-order elimination model. In our study, the assessment of both one- and two-compartment models, together with permutation of other model parameters (Figure S2), showed marginally superior fitting with a one-compartment model, first-order elimination model over other tested models.

The presence of a secondary C_max_ of plasma CNA in both D1C and D2C of mixed spices is an unexpected and novel finding in humans. While this phenomenon was not observed in an i.v. human clinical study [44], similar secondary peaks was observed in rats with oral administration of Cinnamomi Ramulus (*Cinnamomum cassia*) [45]. Several reasons could account for this observation, including enterohepatic recycling, site-specific absorption or delayed gastric emptying. The role of enterohepatic recycling of CNA may be minor, as CNA is primarily eliminated (63%) via oxidation (β-oxidation) and subsequently glycine conjugation to form HA, with a small fraction being glucuronidated only when the liver’s capacity for glycine conjugation is exceeded [41]. Site-specific absorption refers to concurrent absorption in the organs such as the stomach, resulting in multiple sites of absorption and secondary peaks in plasma concentration-time profiles. While CNA may be partially absorbed via monocarboxylic acid transporters (MCT) along the entire length of gastrointestinal tract, CNA shows far greater absorption in duodenum, jejunum, cecum and colon relative to the stomach in rats [46]. Lastly, gastric emptying controls the amount of chyme entering the duodenum and is likely to modulate the absorption of xenobiotics with high solubility and permeability such as CNA. Furthermore, some of the ingredients used in our study (coriander, cumin, garlic, and onion) have been shown to reduce gastric transit time [47], and may therefore affect the kinetics of well-absorbed compounds such as CNA in yielding multiple peaking. It should be noted that secondary peaks in CNA were only observed in 14 out of 20 participants who took part in our study, which further highlighted the inter-individual variability in polyphenol metabolism.

While our study sheds novel insights on the dose-exposure associations between polyphenol rich spice intake and the concentrations of free/unconjugated phenolic/aromatic acids in plasma, there were a few limitations that need to be considered. Apart from measuring the total polyphenol content of the spice containing test meals, we did not characterize nor quantify the individual polyphenols present in these meals. This was principally an analytical hurdle for the team, given the lack of high-resolution mass spectrometry instruments. Furthermore, the treatment of samples with glucuronidase/sulfatase enzymes would have yielded quantification of total aglycones, although for reasons discussed above, we decided against this approach. The study of downstream metabolites of the terminal phenolic acid pathway could potentially be confounded by alternative sources of polyphenols. While the volunteers were asked to avoid polyphenol rich foods for a 72 h prior to study, baseline (0 h) levels of the several metabolites were rather variable, including some having somewhat higher baseline concentrations of certain phenolic acids. This may indicate lower compliance during the 3-day ‘run-in’ periods for some volunteers, who may have consumed these polyphenol-containing foods due to the omnipresence of polyphenols in a plethora of food options. Challenges such as these are inevitable when conducting dietary intervention trials in a free-living population. Similar challenges are now being dealt with by using stable isotopes of polyphenols to accurately understand nutrikinetics and metabolism of polyphenols [48]. Another limitation of this study was the measurement of plasma samples at regular intervals only for a period of up to 7 h. While this is common for metabolic studies in nutrition, this approach may have missed out on further changes in phenolic/aromatic acid concentrations, particularly involving gut mediated metabolism beyond the duration measured. Despite these limitations, our study is a first dose-response study with mixed spices exploring nutrikinetics of plasma phenolic and aromatic acids. The strong dose-exposure association of CNA indicates its potential use as a biomarker of spice intake. We also confirmed findings from the existing literature of the large inter-individual variability in the metabolism of polyphenols.

## Figures and Tables

**Table 1:** Plasma nutrikinetics of terminal aromatic acid pathway metabolites after consumption of control 0 g (D0C), 6 g (D1C), and 12 g (D2C) of mixed spices. C_max_, normalized maximum concentration obtained in plasma (nM); T_max_, time (h) when C_max_ was reached; AUC_0–7 h_, normalized area under the curve over time (nM.h) until 7 h; N.D., not detected. Results are expressed as mean ± standard deviation (*n* = 20 for D0C and D2C; *n* = 17 for D1C). * One outlier (Figure S3) was omitted from nutrikinetic analysis of cinnamic acid.

Nutrikinetic Parameters	C_max_ (nM)			T_max_ (h)			AUC_0–7 h_ (nM.h)		
Polyphenols Intake	D0C	D1C	D2C	D0C	D1C	D2C	D0C	D1C	D2C
Benzoic acids									
Benzoic acid (BA)	114.3 ± 36.7	113.3 ± 33.6	109.0 ± 34.6	5.3 ± 2.2	5.7 ± 2.0	3.8 ± 2.8	347.9 ± 86.8	347.0 ± 107.2	354.7 ± 122.8
3-Hydoxybenzoic acid (3OH-BA)	N.D.	N.D.	N.D.	N.D.	N.D.	N.D.	N.D.	N.D.	N.D.
4-Hydroxybenzoic acid (4OH-BA)	13.7 ± 9.9	18.3 ± 12.4	41.5 ± 101.2	3.1 ± 1.7	3.4 ± 2.1	3.5 ± 1.5	36.0 ± 23.3	54.2 ± 34.6	114.1 ± 207.0
Phenylacetic acids									
Phenylacetic acid (PAA)	433.3 ± 286.9	1019.1 ± 825.9	1001.4 ± 666.2	3.4 ± 2.4	3.6 ± 1.4	3.2 ± 1.3	1742.3 ± 1243.4	4437.2 ± 2974.3	4132.1 ± 2947.1
3-Hydroxyphenylacetic acid (3OH-PAA)	28.1 ± 26.2	27.2 ± 15.8	29.1 ± 20.5	4.9 ± 2.0	4.7 ± 2.6	5.7 ± 1.7	101.0 ± 112.2	105.7 ± 70.0	113.3 ± 90.4
4-Hydroxyphenylacetic acid (4OH-PAA)	565.9 ± 322.3	667.3 ± 304.9	633.4 ± 285.1	4.7 ± 2.0	4.4 ± 2.0	3.8 ± 1.8	2070.7 ± 1453.7	2378.0 ± 1065.8	2166.7 ± 1067.5
Phenylpropanoic acids									
3-Phenylpropanoate acid (PPA)	278.7 ± 322.4	730.7 ± 1224.0	554.4 ± 984.6	2.5 ± 1.8	2.8 ± 2.3	3.0 ±2.2	831.1 ± 783.9	2390.8 ± 4371.0	1859.8 ± 3426.7
3-(3-Hydroxyphenyl)-propanoic acid (3OH- PPA)	58.9 ± 39.1	83.3 ± 79.5	72.5 ± 48.6	3.6 ± 2.8	4.0± 2.8	3.5 ± 2.9	182.2 ± 126.3	283.0 ± 388.2	226.3 ± 176.6
3-(4-Hydroxyphenyl)-propanoic acid (4OH-PPA)	31.4 ± 25.0	48.6 ± 36.6	81.7 ± 102.2	3.1 ± 2.7	2.6 ± 1.7	3.3 ± 2.0	126.3 ± 115.1	178.0 ± 120.3	260.6 ± 131.7
Hippuric acids									
Hippuric acid (HA)	1286.2 ± 706.5	1369.2 ± 1167.5	1075.8 ± 615.7	2.4 ± 3.3	3.4 ± 3.2	0.5 ± 0.3	1832.7 ± 1421.5	3224.5 ± 3095.2	3063.1 ± 1795.5
3-Hydroxyhippuric acid (3OH-HA)	26.3 ± 22.5	27.4 ± 19.1	31.3 ± 26.5	3.3 ± 3.1	4.8 ± 2.9	3.8 ± 3.2	70.7 ± 61.6	80.1 ± 68.3	88.4 ± 85.4
4-Hydroxyhippuric acid (4OH-HA)	N.D.	N.D.	N.D.	N.D.	N.D.	N.D.	N.D.	N.D.	N.D.
Cinnamic acids									
Cinnamic acid (CNA) *	2.4 ± 0.7	164.4 ± 47.7	342.0 ± 83.0	2.8 ± 1.9	0.7 ± 0.4	1.4 ± 1.2	8.4 ± 3.4	376.1 ± 104.7	875.7 ± 291.9

On each of 3 test sessions, volunteers consumed in one of three test meals: Dose 0 Control (D0C), Dose 1 Curry (D1C) and Dose 2 Curry (D2C), containing 0 g, 6 g, and 12 g mixed spices respectively.

## Supplementary Materials

Supplementary materials are available online at http://www.mdpi.com/2072-6643/10/7/934/s1. Figure S1: Proposed polyphenol metabolism pathways. Figure S2: Scatter plot of studied metabolites across all three meal doses. Figure S3: Plasma concentration against time plots of cinnamic acid across all three doses. Table S1: List of polyphenols in the spices and vegetables used in study. Table S2: Mass spectrometry and method validation parameters. Table S3: Nutrikinetic models selection with Akaike information criterion.
