# Supplementary material for: Dose-Dependent Increase in Unconjugated Cinnamic Acid Concentration in Plasma Following Acute Consumption of Polyphenol Rich Curry in the Polyspice Study
Source: Nutrients. 2018 Jul 20;10(7):934. doi: 10.3390/nu10070934 (PMC6073423; doi:10.3390/nu10070934)
Supplement: Supplementary file 1 [file nutrients-10-00934-s001.pdf]

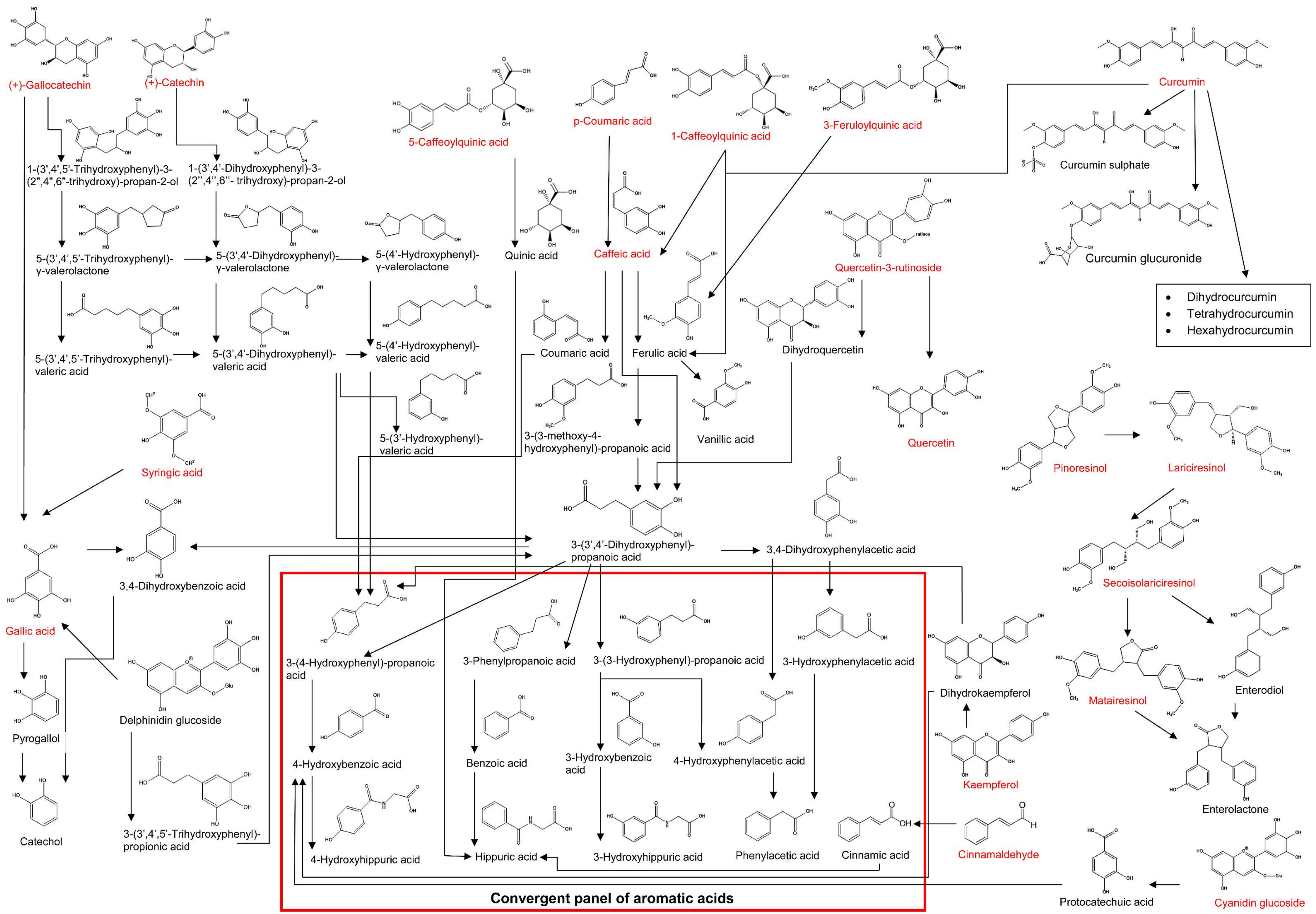

Figure S1 Proposed polyphenol metabolism pathways. Red fonts reflect the parent polyphenols of mixed spices used in this study, while the red rectangle shows the aromatic acids quantified in this study.

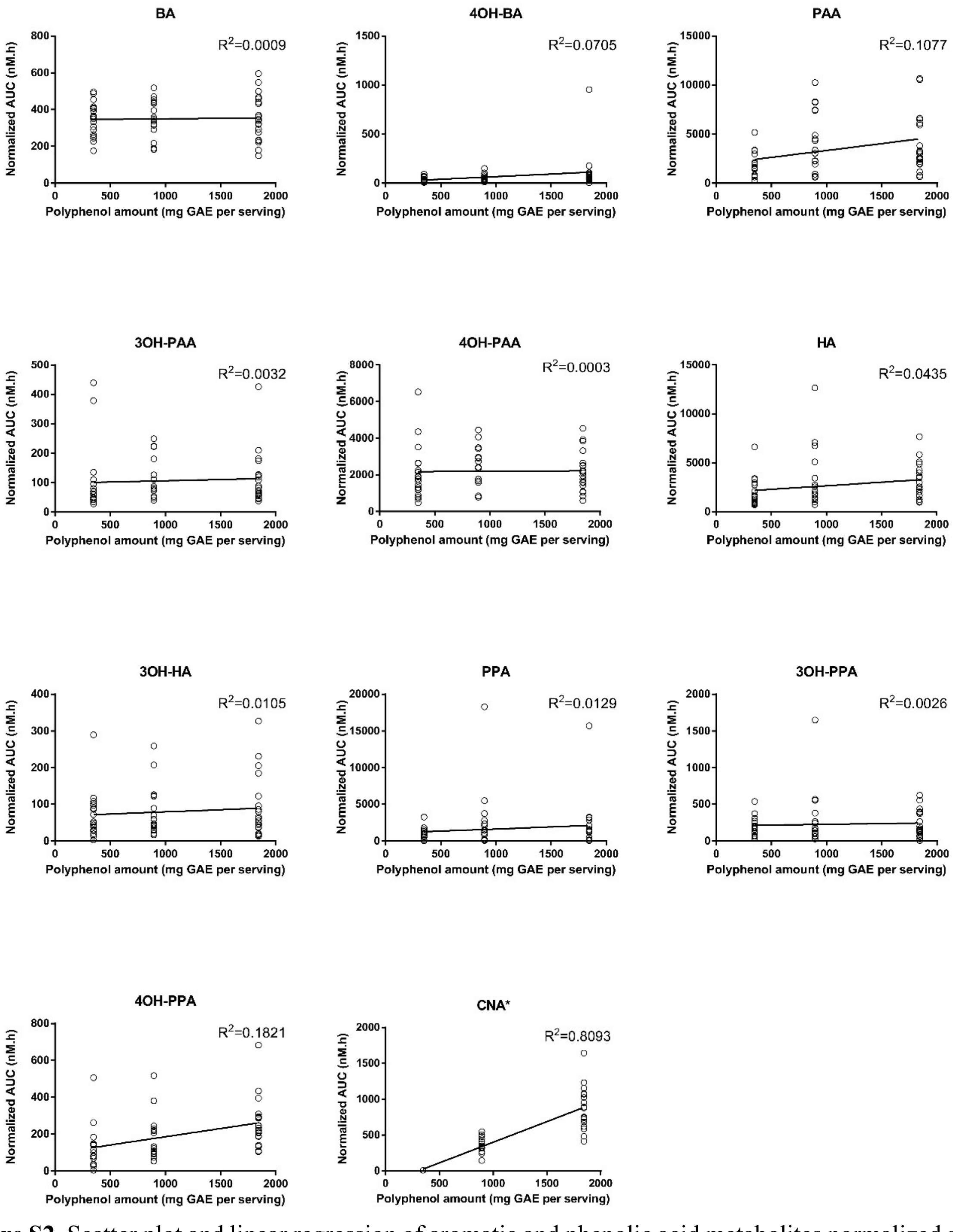

Figure S2. Scatter plot and linear regression of aromatic and phenolic acid metabolites normalized area under the curve (AUC) (nM.h) against total polyphenol content of D0C, D1C and D2C meals (mg of gallic acid equivalent (GAE) per serving) as used in the mixed spices intervention study. Benzoic acid (BA); 4-hydroxybenzoic acid (4OH-BA); phenylacetic acid (PAA); 3-hydroxyphenylacetic acid (3OH-PAA); 4-hydroxyphenylacetic acid (4OH-PAA); 3-phenylpropanoic acid (PPA); 3-(3-hydroxyphenyl)-propanoic acid (3OH-PPA); 3-(4-hydroxyphenyl)-propanoic acid (4OH-PPA), hippuric acid (HA); 3-hydroxyhippuric acid (3OH-HA); and cinnamic acid (CNA); *One outlier was omitted from the regression.

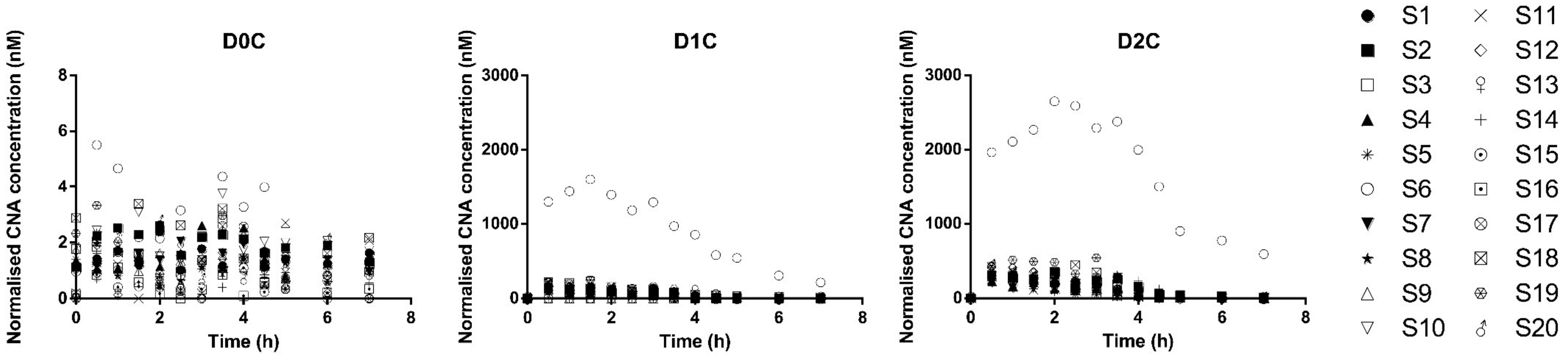

Figure S3. Normalized linear plasma concentration against time plots of cinnamic acid (CNA) across all three doses. One participant was identified as a biological outlier, displaying strong dose-exposure relationship within himself, but higher exposure relative to other participants in the study.

Table S1. List of documented polyphenols and their abundances found in the spices and vegetables used in the study. Mean polyphenol content and total polyphenol content (TPC, based on Folin assay) retrieved from Phenol-Explorer 3.6 (http://phenol-explorer.eu/) and expressed in mg/100mg fresh weight (FW).

Food	Food form	Polyphenol class	Polyphenol sub-class	Polyphenol	Mean content (mg/100g FW)	TPC (mg/100g FW)
**Turmeric**	Turmeric dried	Other polyphenols	Curcuminoids	Bisdemethoxycurcumin	1237.5	
				Curcumin	2213.57	
				Demethoxycurcumin	1982.5	
						2117
**Cumin**	Cumin	Flavonoids	Flavonols	Kaempferol	38.6	
			Flavanones	Eriodictyol	0.3	
			Flavones	Luteolin	3.1	
		Phenolic acids	Hydroxycinnamic acid	Caffeic acid	16.6	
				Ferulic acid	5.8	
			Hydroxybenzoic acids	Gallic acid	1.2	
						2038.33
**Coriander**	Coriander, seed					357.36
**Cinnamon**	Ceylan cinnamon	Phenolic acids	Hydroxybenzoic acids	2-Hydroxybenzoic acid	0.7	
				Protocatechuic acid	1.16	
				Syringic acid	0.78	
			Hydroxycinnamic acids	Caffeic acid	24.2	
				p-Coumaric acid	0.55	
						9700
**Clove**	Cloves	Flavonoids	Flavonols	Kaempferol	23.8	
				Quercetin	28.4	
		Other polyphenols	Hydroxyphenylpropenes	Acetyl eugenol	2075.1	
				Eugenol	12592.93	
		Phenolic acids	Hydroxybenzoic acids	Gallic acid	458.19	
				Protocatechuic acid	0.52	
				Syringic acid	0.79	
				p-Coumaric acid	8.49	
						16047.5
**Cayenne pepper**	Cayenne pepper, raw	Flavonoids	Flavones	Luteolin	1.89	
			Flavonols	Quercetin	2.92	
**Gooseberry (amla)**	Gooseberry	Flavonoids	Anthocyanins	Cyanidin 3-O-(6"-caffeoyl-glucoside)	0.07	
				Cyanidin 3-O-(6"-p-coumaroyl-glucoside)	0.23	
				Cyanidin 3-O-galactoside	1.18	
				Cyanidin 3-O-glucoside	0.005	
				Cyanidin 3-O-rutinoside	2.95	
				Peonidin 3-O-glucoside	2.03	
				Peonidin 3-O-rutinoside	0.06	
			Flavonols	(+)-Catechin	0.1	
				(+)-Gallocatechin	1.67	
				Kaempferol	0.44	
				Quercetin	0.88	
		Lignans	Lignans	Secoisolariciresinol	1.23	
		Phenolic acids	Hydroxybenzoic acids	4-Hydroxybenzoic acid 4-O-glucoside	1.15	
				Protocatechuic acid 4-O-glucoside	0.45	
			Hydroxycinnamic acids	3-Caffeoylquinic acid	0.35	
				3-Feruloylquinic acid	0.1	
				3-p-Coumaroylquinnic acid	0.05	
				Caffeic acid 4-O-glucoside	0.2	
				Caffeoyl glucose	0.9	
				Ferulic acid 4-O-glucoside	0.3	
				p-Coumaric acid 4-O-glucoside	0.5	
				p-Coumaroyl glucose	0.7	
						470.14
**Garlic**	Garlic fresh	Lignans	Lignans	Lariciresinol	17.19	
				Matairesinol	0.00248	
				Pinoresinol	12.1	
				Secoisolariciresinol	1.55	
						87.04
**Ginger**	Ginger, fresh	Lignans	Lignans	Secoisolariciresinol	0.02	
						204.66
**Onion**	Onion (red), raw	Flavonoids	Anthocyanins	Cyanidin 3-O-(6"-malonyl-3"-glucosyl-glucoside)	1	
				Cyanidin 3-O-(6"malonyl-glucoside)	1.5	
				Delphinidin 3-O-glucosyl-glucoside	6.5	
			Flavonols	Isorhamnetin	1.51	
				Isorhamnetin 4'-O-glucoside	6	
				Quercetin	1.31	
				Quercetin 3,4'-O-diglucoside	77.08	
				Quercetin 3-O-glucoside	1.8	
				Quercetin 3-O-rutinoside	0.21	
				Quercetin 4'-O-glucoside	38.8	
				Quercetin 7,4'-O-diglucoside	1.8	
				Kaempferol	0.99	
				Myricetin	0.46	
			Flavones	Apigenin	0.3	
				Luteolin	0.16	
		Phenolic acids	Hydroxybenzoic acids	Protocatechuic acid	2	
						102.83
**Tomato**	Tomato, whole, raw	Flavonoids	Flavanones	Naringenin 7-O-glucoside	0.14	
				Naringenin	0.96	
			Flavonols	Kaempferol	0.01	
				Quercetin	0.00423	

Food	Food form	Polyphenol class	Polyphenol sub-class	Polyphenol	Mean content (mg/100g FW)	TPC (mg/100g FW)
				Quercetin 3-O-rutinoside	0.14	
				Myricetin	0.04	
			Flavones	Apigenin	0.09	
		Lignans	Lignans	Lariciresinol	2.1	
				Matairesinol	0.00000833	
				Medioresinol	0.0035	
				Pinoresinol	0.7	
				Secoisolariciresinol	0.05	
				Syringaresinol	0.0045	
		Phenolic acids	Hydroxycinnamic acids	4-Caffeoylquinic acid	1.17	
				5-Caffeoylquinic acid	1.84	
				Caffeic acid	0.45	
				Ferulic acid	0.27	
				p-Coumaric acid	0.13	
			Hydroxybenzoic acids	4-Hydroxybenzoic acid	0.05	
				Vanillic acid	0.02	
						45.06
**Aubergine**	Aubergine, peeled	Phenolic acid	Hydroxybenzoic acids	4-Hydroxybenzoic acid	0.09	
				Gallic acid	0.14	
				Protocatechuic acid	0.58	
			Hydroxycinnamic acids	Caffeic acid	0.38	
				Ferulic acid	0.22	
				p-Coumaric acid	0.08	

Table S2. Mass spectrometry compound-dependent parameters and method validation parameters, linearity (R^2), accuracy (percentage deviation from spiked), and precision (relative SD). Entrance potential was set at 10 V for all analytes. CE, collision energy; CXP, collision exit potential; DP, declustering potential; Q1, Parent mass/charge; Q3, product mass/charge; RT, retention time.

Polyphenols	Q1	Q3	RT (min)	Dwell (msec)	DP	CE	CXP	Dynamic range (nM)	Linearity R²	Spiked amount (nM)	Accuracy (%)	Precision (%)
**Benzoic acid derivatives**												
Benzoic acid (BA)	121	77	2.52	50	-10	-14	-5	5 – 1000	0.988	50.00	109.79	39.08
										400.00	99.08	8.53
										800.00	93.91	10.20
3-Hydoxybenzoic acid (3OH-BA)	136.7	93	1.8	50	-55	-16	-5	5 – 1000	0.996	50.00	88.42	9.22
										400.00	97.53	4.65
										800.00	98.33	5.34
4-Hydroxybenzoic acid (4OH-BA)	136.7	93.1	1.47	50	-55	-20	-9	5 – 1000	0.995	50.00	87.85	4.90
										400.00	95.99	2.13
										800.00	109.98	7.28
**Phenylacetic acid derivatives**												
Phenylacetic acid (PAA)	134.8	91.1	2.58	50	-45	-10	-13	25 – 5000	0.998	250.00	101.89	12.93
										2000.00	99.27	2.89
										4000.00	93.18	7.00
3-Hydroxyphenylacetic acid (3OH-PAA)	150.8	107	1.88	50	-45	-12	-5	25 – 5000	0.998	250.00	101.45	5.38
										2000.00	99.07	1.20
										4000.00	93.75	6.02
4-Hydroxyphenylacetic acid (4OH-PAA)	150.8	107	1.66	50	-45	-10	-9	25 – 5000	0.995	250.00	106.01	11.41
										2000.00	96.82	5.96
										4000.00	95.18	7.79
**Phenylpropanoate derivatives**												
3-Phenylpropanoate acid (PPA)	148.8	105	2.98	50	-55	-14	-9	25 – 5000	0.997	250.00	99.43	6.39
										2000.00	97.27	2.84
										4000.00	94.02	8.29
3-(3-Hydroxyphenyl)-propanoic acid (3OH-PPA)	164.7	106	2.27	50	-65	-30	-15	5 – 1000	0.995	50.00	98.40	17.09
										400.00	98.66	2.23
										800.00	97.95	8.59
3-(4-Hydroxyphenyl)-propanoic acid (4OH-PPA)	164.7	59	2.1	50	-60	-16	-7	5 – 1000	0.996	50.00	107.23	16.38
										400.00	99.99	5.16
										800.00	95.36	7.61
**Hippuric acid derivatives**												
Hippuric acid (HA)	177.7	134	1.56	50	-50	-16	-11	25 – 5000	0.993	250.00	95.69	18.54
										2000.00	100.92	3.31
										4000.00	96.68	11.88
3-Hydroxyhippuric acid (3OH-HA)	193.7	149.9	0.97	50	-70	-18	-11	5 – 1000	0.996	50.00	95.07	7.54
										400.00	94.43	5.59
										800.00	92.93	9.88
4-Hydroxyhippuric acid (4OH-HA)	193.8	99.9	0.85	50	-50	-14	-9	5 – 1000	0.995	50.00	77.08	12.81
										400.00	91.89	3.94
										800.00	97.45	7.78
**Cinnamic acid derivatives**												
Cinnamic acid (CNA)	146.7	103	2.99	50	-40	-14	-9	5 – 1000	0.998	50.00	100.33	7.10
										400.00	98.08	6.55
										800.00	94.42	5.91

Table S3. Comparison of various nutrikinetic models with the Akaike information criterion. Akaike information criterion is a measure of relative quality of statistical models between a set of model parameters, with low value denoting better model fit within each weighting (1/Y, 1/Y^2, no weighting). K_a, 1st order absorption rate constant; K_e, 1st order elimination rate constant; N.A., not applicable.

					**AIC value**		
**Weighting**	**Compartment**	**Rate constant**	**Lag time**	**Elimination rate**	**Dose 0**	**Dose 1**	**Dose 2**
1/Y^2	1	-	No	1st order	29.58	51.88	10.50
1/Y^2	1	-	Yes	1st order	31.54	53.82	12.48
1/Y^2	1	K_e=K_a	No	1st order	32.85	50.23	8.51
1/Y^2	1	K_e=K_a	Yes	1st order	N.A.	52.23	10.51
1/Y^2	2	Micro	No	1st order	N.A.	55.12	14.50
1/Y^2	2	Micro	Yes	1st order	N.A.	57.07	16.47
1/Y	1	-	No	1st order	26.66	77.05	76.55
1/Y	1	-	Yes	1st order	28.58	75.98	77.66
1/Y	1	K_e=K_a	No	1st order	31.30	75.24	74.55
1/Y	1	K_e=K_a	Yes	1st order	33.30	77.24	76.55
1/Y	2	Micro	No	1st order	N.A.	N.A.	80.55
1/Y	2	Micro	Yes	1st order	N.A.	80.02	81.66
No weighting	1	-	No	1st order	23.66	100.83	127.14
No weighting	1	-	Yes	1st order	25.54	97.82	124.51
No weighting	1	K_e=K_a	No	1st order	29.89	106.52	127.45
No weighting	1	K_e=K_a	Yes	1st order	N.A.	N.A.	N.A.
No weighting	2	Micro	No	1st order	N.A.	104.83	131.13
No weighting	2	Micro	Yes	1st order	N.A.	101.87	128.50
